# NQO1-induced activation of AMPK contributes to cancer cell death by oxygen-glucose deprivation

**DOI:** 10.1038/srep07769

**Published:** 2015-01-14

**Authors:** Hyemi Lee, Eun-Taex Oh, Bo-Hwa Choi, Moon-Taek Park, Ja-Kyeong Lee, Jae-Seon Lee, Heon Joo Park

**Affiliations:** 1 Department of Microbiology, Inha Research Institute for Medical Sciences, College of Medicine, Inha University, Incheon, Korea; 2 Hypoxia-related Disease Research Center, College of Medicine, Inha University, Incheon, Korea; 3 Pohang Center for Evaluation of Biomaterials, Pohang Technopark, Pohang, Gyeongbuk, Korea; 4 Research Center, Dongnam Institute of Radiological & Medical Sciences (DIRAMS), Busan, Korea; 5 Department of Anatomy, College of Medicine, Inha University, Incheon, Korea; 6 Department of Biomedical Sciences, College of Medicine, Inha University, Incheon, Korea

## Abstract

Oxygen and glucose deprivation (OGD) due to insufficient blood circulation can decrease cancer cell survival and proliferation in solid tumors. OGD increases the intracellular [AMP]/[ATP] ratio, thereby activating the AMPK. In this study, we have investigated the involvement of NQO1 in OGD-mediated AMPK activation and cancer cell death. We found that OGD activates AMPK in an NQO1-dependent manner, suppressing the mTOR/S6K/4E-BP1 pathway, which is known to control cell survival. Thus, the depletion of NQO1 prevents AMPK-induced cancer cell death in OGD. When we blocked OGD-induced Ca^2+^/CaMKII signaling, the NQO1-induced activation of AMPK was attenuated. In addition, when we blocked the RyR signaling, the accumulation of intracellular Ca^2+^ and subsequent activation of CaMKII/AMPK signaling was decreased in NQO1-expressing cells under OGD. Finally, siRNA-mediated knockdown of CD38 abrogated the OGD-induced activation of Ca^2+^/CaMKII/AMPK signaling. Taken together, we conclude that NQO1 plays a key role in the AMPK-induced cancer cell death in OGD through the CD38/cADPR/RyR/Ca^2+^/CaMKII signaling pathway.

Within solid tumors, subpopulations of cancer cells are exposed to very low oxygen (hypoxic) and glucose conditions due to inadequate perfusion of blood through poorly developed and heterogeneous vascular beds^1^. Oxygen and glucose deprivation (OGD) causes metabolic stresses that interfere with ATP, increasing the [AMP]/[ATP] ratio and activating AMP-activated protein kinase (AMPK)^2^.

AMPK is a highly conserved metabolic sensor belonging to a class of serine/threonine kinases that are sensitive to various environmental stresses, especially those that perturb the cellular energy status^3^,^4^,^5^. It is a heterotrimeric complex consisting of a catalytic α subunit, a scaffolding β subunit, and a regulatory γ subunit, all of which are encoded by distinct genes (α1 and α2; β1 and β2; and γ1, γ2, and γ3, respectively)^4^,^6^,^7^. When cellular energy levels are decreased (and thus the AMP/ATP ratio is increased), AMPK is phosphorylated by liver kinase B1 (LKB1)^7^,^8^,^9^. However, Ca^2+^/calmodulin-dependent kinase has also been identified as an AMPK kinase^7^,^8^,^9^,^10^. Activated AMPK can phosphorylate diverse targets involved in controlling cellular energy metabolism; it switches on catabolic pathways that generate ATP while switching off anabolic pathways^2^,^11^. In catabolic pathways, AMPK activates glucose uptake via glucose transporters (GLUT1 and GLUT4) and increases glycolysis^2^. In anabolic pathways, AMPK targets acetyl coenzyme A carboxylases 1 and 2 (ACC1/2), which synthesize malonyl-CoA^2^,^4^. Importantly, diverse target proteins of AMPK have recently been reported to be involved in regulating cancer cell death or survival. For example, AMPK has been shown to inhibit the proliferation and survival of cancer cells by suppressing mTOR (mammalian target of rapamycin) and its downstream regulators, S6K (ribosomal protein S6 kinase) and 4E-BP1 (eukaryotic initiation factor 4E binding protein 1)^12^,^13^. AMPK also inhibits cell proliferation by increasing the cellular levels of p21 and p53^14^. In contrast, however, AMPK activation has also been reported to suppress apoptosis of endothelial cells by increasing the nuclear factor-kappa B (NF-κB)-mediated expression of the anti-apoptotic proteins, Bcl-2 and survivin^15^.

NAD(P)H:quinone oxidoreductase 1 (NQO1) is a cytosolic reductase that plays important roles in the cellular response to numerous stresses, and is up-regulated in many human cancers compared to adjacent normal tissues^16^,^17^,^18^. Upregulation of NQO1 has been shown to protect cells against various cytotoxic quinones and oxidative stress by catalyzing the reduction and detoxification of quinine substrates^19^,^20^,^21^. For example, NQO1-null mice exhibit increased susceptibility to chemical-induced skin carcinogenesis via abrogation of NF-κB, Akt, and mitogen-activated protein kinases^22^,^23^,^24^. In addition, upregulation of NQO1 was shown to protect cell from ischemia-reperfusion injury^25^. On the other hand, it has been reported that NQO1 act as a pro-apoptotic factor. For instance, blockade of NQO1 inhibits p53-mediated apoptosis in γ-irradiated thymocytes and reduces neuronal damage^26^,^27^. At the present, the mechanisms underlying these opposing effects of NQO1 on cell survival and death remain controversial. The expression of NQO1 is elevated by hypoxia/reoxygenation or inflammatory stresses through nuclear accumulation of the NQO1 transcription factor, Nrf2 (NFE2-related factor 2)^25^,^28^. Activation of the cytoprotective Nrf2 antioxidant pathway by sulforaphane protects immature neurons and astrocytes from death caused by exposure to combined hypoxia and glucose deprivation^29^,^30^.

The purpose of the present study was to investigate the involvement of NQO1 in the activation of AMPK and the suppression of mTOR in cancer cells under oxidative stress (more specifically, OGD). Here, we report for the first time that OGD activates AMPK through the CD38/cADPR/RyR/Ca^2+^/CaMKII signaling pathway, leading to the NQO1-dependent inactivation of mTOR. Our results clearly demonstrate that NQO1 plays a key role in OGD-induced cell death, and that this occurs via the activation of AMPK.

## Results

### The activation of AMPK by OGD depends on NQO1

**Figure 1:**
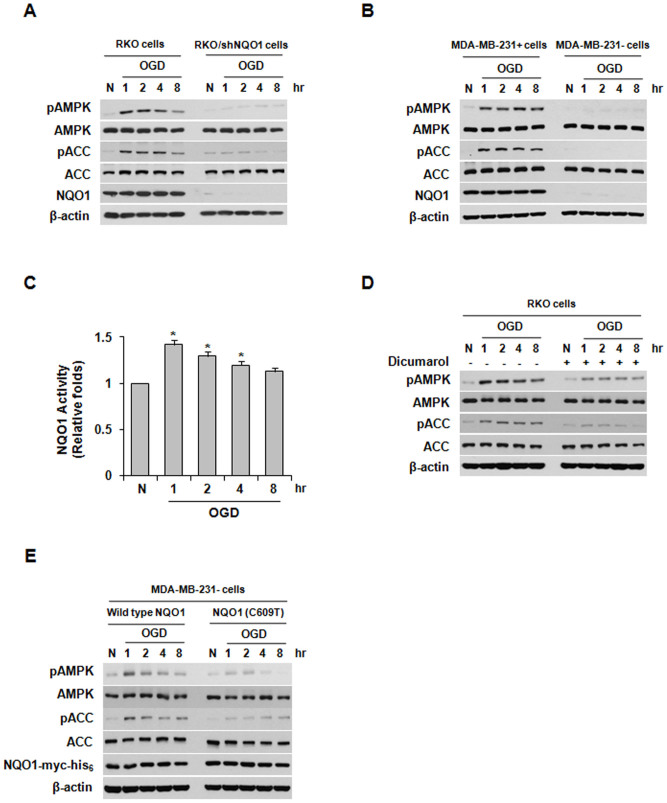
The involvement of NQO1 in AMPK activation following oxygen and glucose deprivation (OGD). (A) Expression of pAMPK, AMPK, pACC, ACC, NQO1, and β-actin in parental RKO and RKO/shNQO1 cells exposed to OGD for the indicated times. (B) Expression of pAMPK, AMPK, pACC, ACC, NQO1, and β-actin in MDA-MB-231+ and MDA-MB-231- cells exposed to OGD for the indicated times. (C) NQO1 activity in RKO cells exposed to OGD for the indicated times. The results from three independent experiments are expressed as means ± SEM (*, P < 0.05). (D) Effect of dicumarol on AMPK activation in RKO cells exposed to OGD. Expression of pAMPK, pACC, AMPK, ACC, and β-actin in RKO cells that were pretreated with dicumarol (5 μM) and exposed to OGD for the indicated times. (E) Expression of pAMPK, pACC, AMPK, ACC, NQO1-myc-His_6_, and β-actin in RKO cells in MDA-MB-231-/pNQO1 and MDA-MB-231-/pNQO1 (C609T) cells exposed to OGD for the indicated times. Abbreviation: N, normoxic control.

It has been reported that AMPK is activated in tumor microenvironments characterized by low oxygen and glucose deprivation^2^. To investigate the effect of NQO1 on AMPK activation in cells undergoing OGD, we exposed parental RKO cells and RKO cells stably transfected with vectors expressing NQO1 shRNA (RKO/shNQO1 cells) to OGD. As shown in Figure 1A, OGD increased the phosphorylations of both AMPK and ACC (acetyl-CoA carboxylase, a well-known downstream target of AMPK) in RKO cells for 1–8 h post-treatment, but not in RKO/shNQO1 cells during this period. To confirm these observations, we assessed the phosphorylation of AMPK in NQO1-deficient MDA-MB-231 cells (MDA-MB-231- cells) stably transfected with an NQO1-encoding expression plasmid. In agreement with the results obtained in RKO cells, NQO1-overexpressing MDA-MB-231 cells (MDA-MB-231+ cells) under OGD showed markedly higher phosphorylation levels of both AMPK and ACC, compared to parental MDA-MB-231- cells under the same conditions (Figure 1B). Consistent with a previous study showing that NQO1 activity is increased by OGD^26^, we found that NQO1 activity significantly increased after exposure of cells to OGD for 1 h, then gradually declined thereafter among cells still under OGD (Figure 1C). To further examine the involvement of NQO1 in the activation of AMPK under OGD, RKO cells were treated with NQO1 inhibitor, dicumarol. Our results revealed that dicumarol effectively inhibited the OGD-induced phosphorylations of AMPK and its target protein, ACC (Figure 1D). To confirm these results, we overexpressed wild-type NQO1 and NQO1 mutated at its known catalytic active site (C609T) in MDA-MB-231- cells, and exposed the cells to OGD. We found that NQO1 activity increased for 1 h under OGD and gradually decreased thereafter in cells expressing wild-type (but not mutant) NQO1 (Figure S1). As shown in Figure 1E, the phosphorylation levels of AMPK and ACC were significantly reduced in NQO1 mutant-expressing cells. Since inhibition of mTOR through activation of the AMPK-TSC2 (tuberous sclerosis complex 2) pathway has been shown to suppress the initiation of mRNA translation and ribosome biogenesis^31^,^32^, we investigated the effect of NQO1 on the activation of mTOR under OGD. As expected, the phosphorylation of mTOR was significantly decreased under OGD in RKO cells expressing wild-type NQO1, but not in NQO1-deficient parental MDA-MB-231 cells (Figure S2A). To further confirm the direct involvement of AMPK in suppressing mTOR under OGD, we transfected RKO cells with siRNAs targeting AMPK and then examined the phosphorylation levels of the mTOR targets, S6K and 4E-BP1. Among cells exposed to OGD for 8 h, siRNA-mediated inhibition of AMPK attenuated the OGD-induced hypophosphorylations of S6K and 4E-BP1 (Figure S2B), suggesting that OGD-induced AMPK activation suppressed mTOR signaling. Together, these data indicate that NQO1 plays an important role in activating the AMPK pathway in cancer cells under OGD.

### CaMKII regulates AMPK activity under OGD in NQO1-dependent manner

As AMP is known to cause allosteric activation of AMPK, thereby rendering it a better substrate for phosphorylation^6^,^33^, we herein studied the effects of NQO1 on the intracellular [AMP]/[ATP] ratio under OGD. In our system, the intracellular [AMP]/[ATP] ratio increased much more in RKO/shNQO1 cells than in parental RKO cells (Figure S3A), even though our earlier experiments had shown that the phosphorylation status of AMPK under OGD was substantially lower in RKO/shNQO1 cells than in parental RKO cells (Figure 1A). These results indicate that NQO1 may positively regulate OGD-induced AMPK activation regardless of the intracellular [AMP]/[ATP] ratio. Since LKB1 (liver kinase B1) has been reported to be an upstream activator of AMPK^6^,^33^, we investigated whether NQO1 is also involved in activating LKB1 under OGD. However, exposure to OGD had little effect on the cellular levels of total or phosphorylated LKB1 in parental or RKO/shNQO1 cells (Figure S3B), suggesting that OGD does not act through LKB1 to induce AMPK activation.

**Figure 2:**
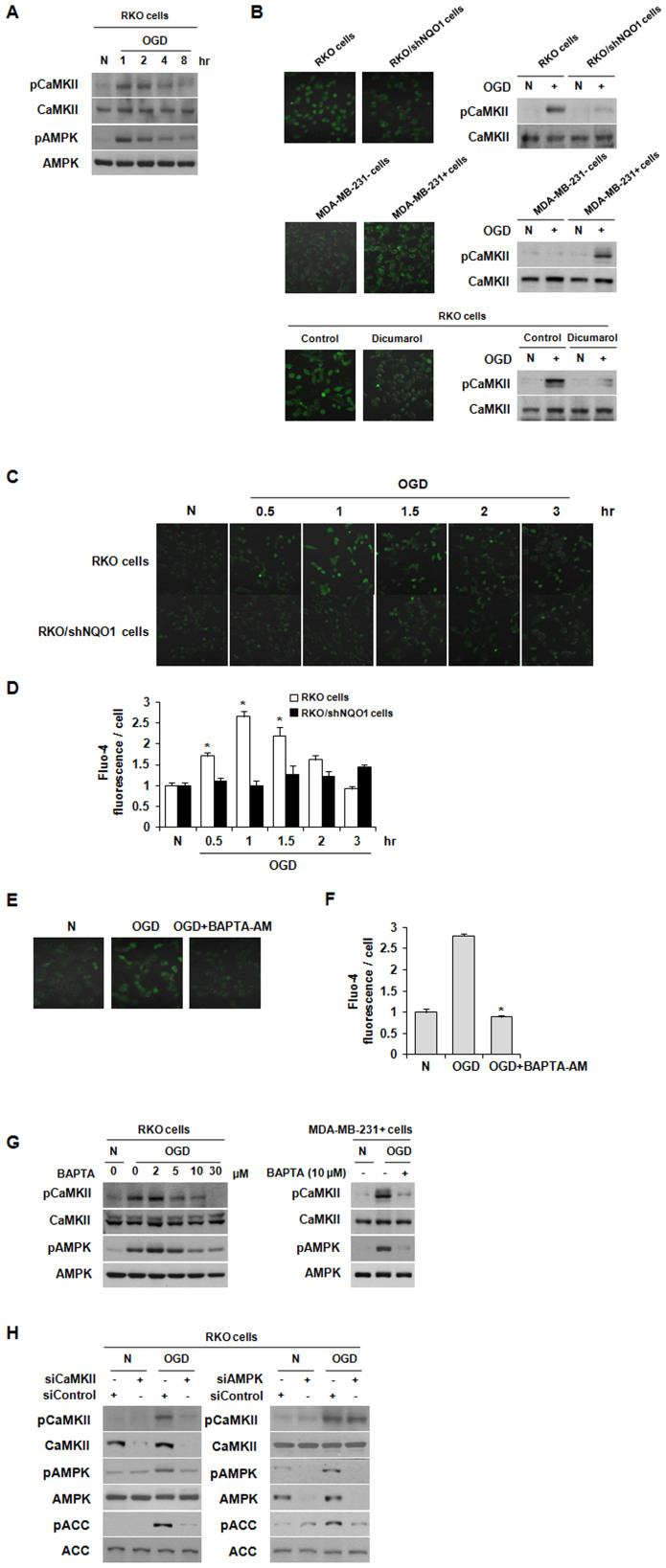
NQO1 activates the CaMKII-AMPK pathway under OGD. (A) Phosphorylation levels of CaMKII and AMPK in RKO cells exposed to OGD for the indicated times. (B) Involvement of NQO1 in OGD-induced elevation of [Ca^2+^]_i_ and activation of CaMKII. Fluo-4-AM-loaded RKO, RKO/shNQO1, MDA-MB-231+ and MDA-MB-231- cells and Fluo-4-AM-loaded RKO cells treated with or without dicumarol were exposed to OGD for 1 h. [Ca^2+^]_i_ was visualized under a fluorescence microscope, and phosphorylation of CaMKII was determined by immunoblotting. (C)-(D) Effect of OGD on [Ca^2+^]_i_ in RKO cells. Fluo-4-AM-loaded cells were exposed to OGD for the indicated times. Representative fluorescence microscopic images (C) and quantification of [Ca^2+^]_i_ (D) are shown. The results from three independent experiments are expressed as means ± SEM (*, P < 0.05). (E)-(F) Effect of BAPTA-AM on Ca^2+^ signaling in RKO cells exposed to OGD. Fluo-4-AM-loaded cells were pretreated with BAPTA-AM (30 μM) and exposed to OGD for 1 h. Representative fluorescence microscopic images (E) and quantification of [Ca^2+^]_i_ (F) are shown. The results from three independent experiments are expressed as means ± SEM (*, P < 0.05). (G) RKO cells pretreated with different concentrations of BAPTA-AM and MDA-MB-231+ cells pretreated with 10 μM of BAPTA-AM were exposed to OGD for 1 h, and whole-cell lysates were analyzed by immunoblotting using antibodies against pCaMKII, pAMPK, CaMKII, and AMPK. (H) RKO cells transfected with siRNAs against AMPK or CaMKII were exposed to OGD for 1 h, and phosphorylation of AMPK, ACC and CaMKII in whole-cell lysates was determined by immunoblotting. Abbreviation: N, normoxic control.

Previous studies have demonstrated that AMPK is also activated by Ca^2+^/calmodulin (CaM)-dependent protein kinase kinase II (CaMKKII) in response to increases in intracellular Ca^2+^ levels ([Ca^2+^]_i_), and that [Ca^2+^]_i_ is increased by hypoxic stimuli in A549 lung cancer cells^7^,^8^,^9^,^10^. Thus, we examined the effect of OGD on the activation of CaMKII in RKO cells. We found that phosphorylation of CaMKII increased during the first 2 h of cellular exposure to OGD, thus correlating with the window of time during which AMPK is activated (Figure 2A). Next, the effect of NQO1 on OGD-induced accumulation of [Ca^2+^]_i_ and phosphorylation of CaMKII was investigated. As shown in Figure 2B, [Ca^2+^]_i_ and CaMKII phosphorylation were both increased in NQO1-expressing cells under OGD, and the dicumarol effectively inhibited OGD-induced increase of [Ca^2+^]_i _and phosphorylation of CaMKII in NQO1-expressing RKO cells. In addition, [Ca^2+^]_i_ peaked 1 h after exposure to OGD and declined thereafter in NQO1-expressing cells (Figure 2C and 2D), whereas inactivation of NQO1 prevented the OGD-induced elevation of [Ca^2+^]_i_ (Figure S4). To further examine whether Ca^2+^ signaling cascades are involved in regulating OGD-induced AMPK activation, we pretreated RKO cells with the Ca^2+^ chelator, BAPTA-AM. As shown in Figure 2E and 2F, BAPTA-AM efficiently prevented the OGD-induced increase in [Ca^2+^]_i_. Furthermore, pretreatment of the cells with BAPTA-AM dose-dependently reduced the OGD-induced phosphorylations of CaMKII and AMPK in RKO cells (Figure 2G). Identical results were observed in MDA-MB-231+ cells treated with 10 μM of BAPTA-AM. To confirm the relationship between CaMKII and AMPK under OGD, we transfected RKO cells with siRNAs targeting CaMKII or AMPK, and then monitored the activation (phosphorylation) of these proteins by immunoblot analysis. As shown in Figure 2H, siRNA-mediated knockdown of CaMKII efficiently attenuated the OGD-induced phosphorylation of AMPK and ACC, whereas siRNA-mediated inhibition of AMPK had no effect on the OGD-induced phosphorylation of CaMKII, suggesting that CaMKII is an upstream activator of AMPK. Taken together, these results indicate that NQO1 activity-mediated increases in [Ca^2+^]_i_ trigger the CAMKII activation and AMPK signaling under OGD.

### The increase of [Ca^2+^]_i_ under OGD requires cADPR

**Figure 3:**
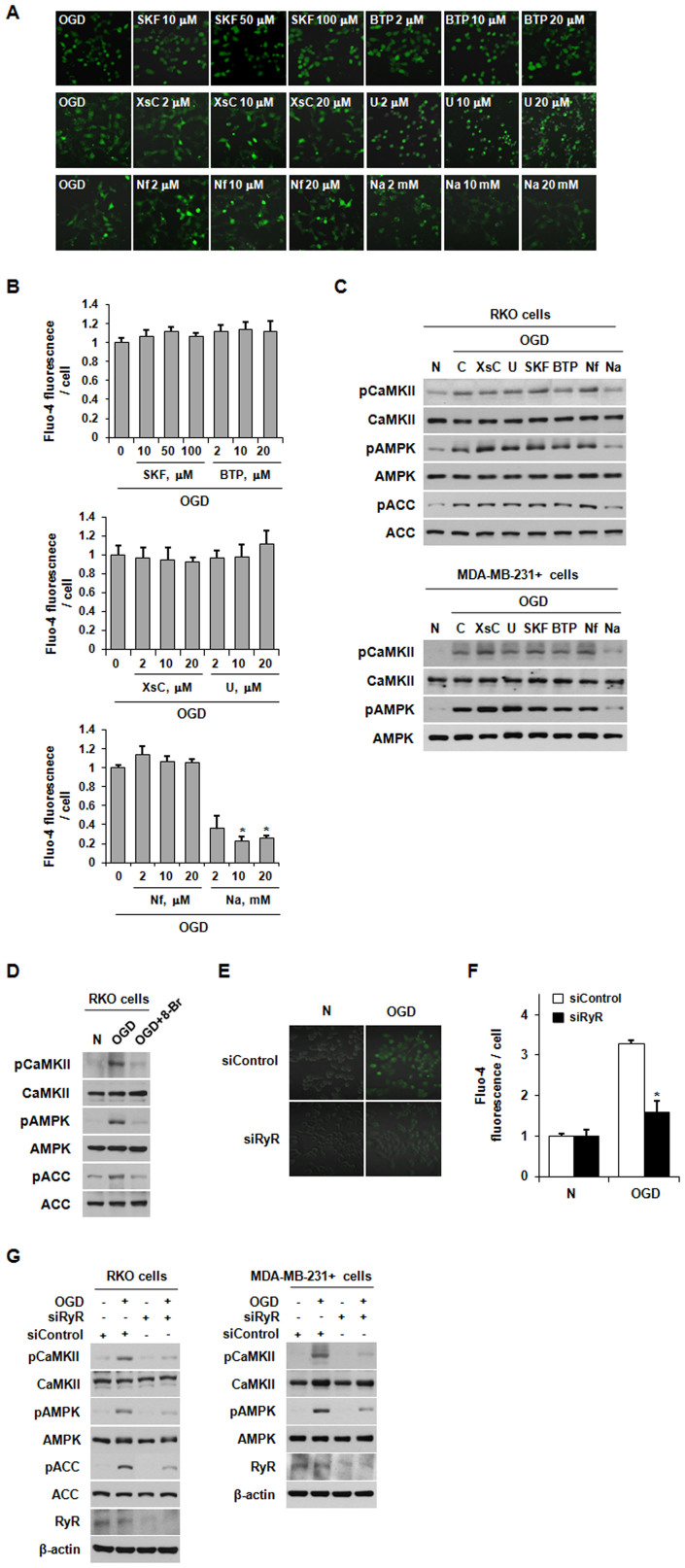
The OGD-induced increase in [Ca^2+^]_i_ requires cADPR. (A)–(B) Effect of Ca^2+^ channel inhibitors on Ca^2+^ signaling in RKO cells exposed to OGD. Fluo-4-AM-loaded cells were pretreated with xestospongin C (XsC), U73122 (U), SKF96365 (SKF), BTP2 (BTP), nifedipine (Nf) or nicotinamide (Na), and exposed to OGD for 1 h. Representative fluorescence microscopic images (A) and quantification of [Ca^2+^]_i_ (B) are shown. (C) RKO and MDA-MB-231+ cells pretreated with the indicated inhibitors were exposed to OGD for 1 h, and whole-cell lysates were analyzed by immunoblotting using antibodies against pCaMKII, pAMPK, pACC, CaMKII, AMPK, ACC. (D) RKO cells pretreated with 8-Br-cADPR were exposed to OGD for 1 h, and whole-cell lysates were analyzed by immunoblotting using antibodies against pCaMKII, pAMPK, pACC, CaMKII, AMPK, and ACC. (E)-(F) Role of RyR in Ca^2+^ signaling among RKO cells exposed to OGD. RKO cells transfected with siRNA against RyR were exposed to OGD for 1 h. Representative fluorescence microscopic images (E) and quantification of [Ca^2+^]_i_ (F) are shown. The results from three independent experiments are expressed as means ± SEM (*, P < 0.05). (G) RKO and MDA-MB-231+ cells transfected with siRNAs against RyR were exposed to OGD for 1 h, and whole-cell lysates were analyzed by immunoblotting using antibodies against pCaMKII, pAMPK, pACC, CaMKII, AMPK and ACC. Abbreviation: N, normoxic control.

Because Ca^2+^ clearly plays a crucial role in the OGD-induced activation of the CaMKII/AMPK pathway in the presence of NQO1, we next examined which Ca^2+^ channels are required for the increase of [Ca^2+^]_i_ observed in cells under OGD. To this end, we pretreated RKO cells with the following: the inositol-trisphosphate receptor inhibitor, xestospongin C; the TRPC (classic transient receptor potential) channel inhibitor, SKF-96365; the CRAC (Ca^2+^ release-activated Ca^2+^) channel inhibitor, BTP-2; or the L-type Ca^2+^ channel inhibitor, nifedipine. We also tested the effects of the phospholipase C inhibitor, U-73122, and the ADP-ribosyl cyclase inhibitor, nicotinamide. As shown in Figure 3A and 3B, pretreatment with nicotinamide substantially inhibited the OGD-induced increase in [Ca^2+^]_i _in NQO1-expressing RKO cells. Subsequently, the nicotinamide efficiently attenuated the OGD-induced phosphorylations of CaMKII and AMPK in NQO1-expressing RKO and MDA-MB-231+ cells (Figure 3C). Notably, the other inhibitors had no such effect, indicating that the ADP-ribosyl cyclase product, cyclic ADP-ribose (cADPR), may play a crucial role in the OGD-induced activation of the CaMKII/AMPK pathway in NQO1-expressing cells (Figure 3C). Since cADPR functions as a potent intracellular second messenger that increases cytosolic Ca^2+^ by binding/activating RyR and promoting the release of Ca^2+^ from intracellular stores^34^, we examined whether RyR contributes to the [Ca^2+^]_i_ increase and CaMKII/AMPK pathway activation observed in NQO1-expressing cells under OGD. As shown in Figure 3D, pretreatment with 8-Br-cADPR, a selective antagonist of cADPR, greatly reduced the OGD-induced activation of the CaMKII/AMPK pathway. We confirmed the specificity of this effect by using siRNAs targeting RyR. Consistent with the results obtained using pharmacological blockers, transfection of RKO cells with RyR-targeting siRNAs effectively attenuated the OGD-induced increase in [Ca^2+^]_i_ (Figure 3E and 3F). Furthermore, knockdown of RyR by siRNA also substantially inhibited the OGD-induced activation of the CaMKII/AMPK pathway in NQO1-expressing RKO and MDA-MB-231+ cells (Figure 3G). These results suggest that OGD induces a cADPR/RyR-mediated increase in [Ca^2+^]_i_ in NQQ1-expressing cells, leading to activation of the CaMKII/AMPK pathway.

### The NQO1-mediated CD38/NAD^+^/cADPR pathway activates the CaMKII/AMPK pathway via accumulation of [Ca^2+^]_i_ under OGD

**Figure 4:**
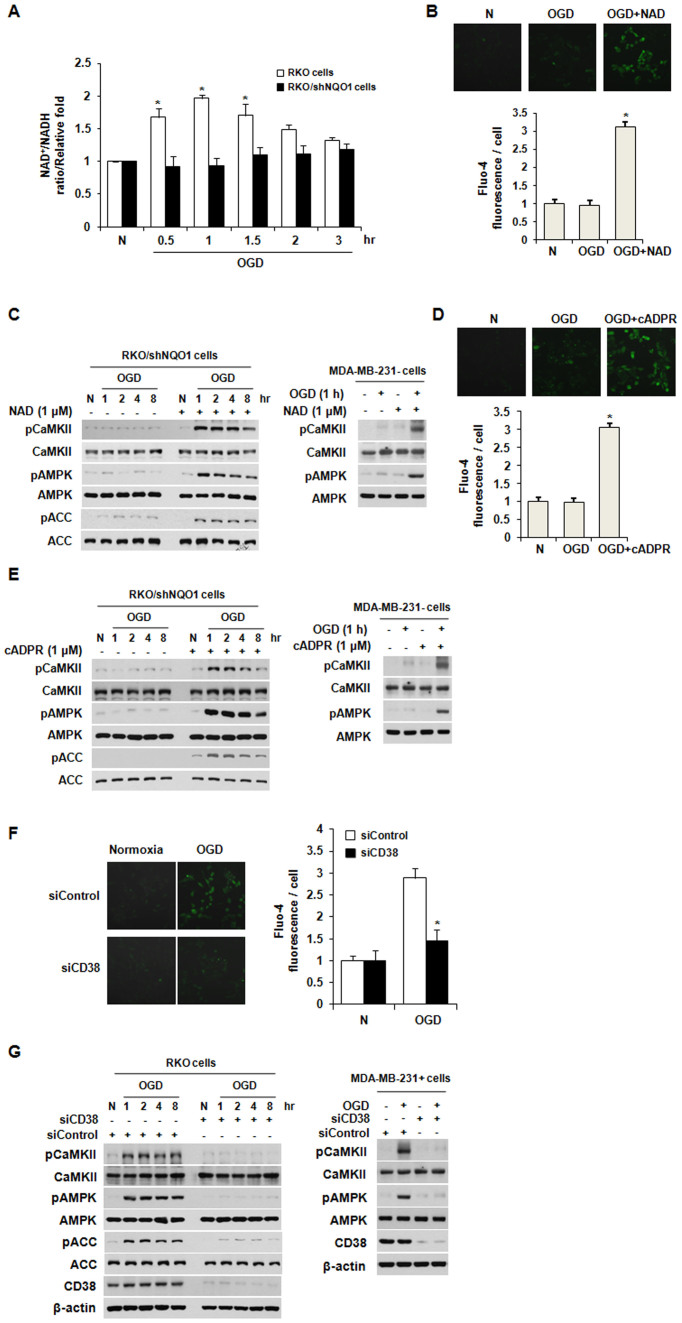
CD38-mediated cADPR production and CaMKII/AMPK pathway activation require NQO1. (A) Intracellular ratio of NAD^+^ to NADH in parental RKO and RKO/shNQO1 cells exposed to OGD for the indicated times. The results from three independent experiments are expressed as means ± SEM (*, P < 0.05). (B) Effect of NAD^+^ on Ca^2+^ signaling in RKO/shNQO1 cells exposed to OGD. Fluo-4-AM-loaded cells were pretreated with NAD^+^ (1 μM) and exposed to OGD for 1 h. Representative fluorescence microscopic images (upper panel) and quantification of [Ca^2+^]_i_ (lower panel) are shown. The results from three independent experiments are expressed as means ± SEM (*, P < 0.05). (C) RKO/shNQO1 and MDA-MB-231- cells pretreated with NAD^+^ (1 μM) were exposed to OGD for the indicated times, and whole-cell lysates were analyzed by immunoblotting using antibodies against pCaMKII, pAMPK, pACC, CaMKII, AMPK, and ACC. (D) Effect of cADPR on Ca^2+^ signaling in RKO/shNQO1 cells exposed to OGD. Fluo-4-AM-loaded cells were pretreated with cADPR (1 μM) and exposed to OGD for 1 h. Representative fluorescence microscopic images (upper panel) and quantification of [Ca^2+^]_i_ (lower panel) are shown. The results from three independent experiments are expressed as means ± SEM (*, P < 0.05). (E) RKO/shNQO1 and MDA-MB-231- cells pretreated with cADPR (1 μM) were exposed to OGD for the indicated times, and whole-cell lysates were analyzed by immunoblotting using antibodies against pCaMKII, pAMPK, pACC, CaMKII, AMPK, and ACC. (F) Involvement of CD38 in Ca^2+^ signaling in RKO cells exposed to OGD. RKO cells transfected with siRNA against CD38 were exposed to OGD for 1 h. Representative fluorescence microscopic images (left panel) and quantification of [Ca^2+^]_i_ (right panel) are shown. The results from three independent experiments are expressed as means ± SEM (*, P < 0.05). (G) RKO and MDA-MB-231+ cells transfected with siRNAs against CD38 were exposed to OGD for indicated times, and whole-cell lysates were analyzed by immunoblotting using antibodies against pCaMKII, pAMPK, pACC, CaMKII, AMPK, ACC, and β-actin. Abbreviation: N, normoxic control.

It has been reported that activation of NQO1 increases the intracellular ratio of NAD^+^ to NADH^35^. Because OGD clearly increased the activity of NQO1 (Figure 1C), we examined whether OGD might increase the ratio of NAD^+^ to NADH. As shown in Figure 4A, OGD increased the ratio of NAD^+^ to NADH in parental RKO cells after 1 h of OGD, but no such effect was seen in RKO/shNQO1 cells. Next, we investigated whether NQO1-mediated NAD^+^ production is required for the [Ca^2+^]_i_-accumulation-mediated production of cADPR and activation of CaMKII/AMPK under OGD. We treated RKO/shNQO1 cells with NAD^+^ and exposed them to OGD for 8 h. Our results revealed that this NAD^+^ treatment efficiently increased intracellular [Ca^2+^]_i_ and activated the CaMKII/AMPK pathway (Figure 4B and 4C). In agreement with the results obtained in RKO/shNQO1 cells, treatment with NAD^+^ activated the CaMKII/AMPK pathway in MDA-MB-231- cells (Figure 4C). To further determine whether cADPR is required for this process, we treated RKO/shNQO1 and MDA-MB-231- cells with cADPR and exposed them to OGD. As shown in Figure 4D and 4E, this cADPR treatment increased [Ca^2+^]_i_ and activated the CaMKII/AMPK pathway in NQO1-deficient cells. Previous studies have demonstrated that the ADP-ribosyl cyclase, CD38, synthesizes cADPR from NAD^35^,^36^,^37^,^38^. To determine the involvement of CD38 in the [Ca^2+^]_i_-mediated activation of the CaMKII/AMPK pathway under OGD, we transfected NQO1-expressing cells with siRNAs targeting CD38 and exposed them to OGD for 8 h. Our results revealed that the inhibition of CD38 completely abrogated the OGD-induced [Ca^2+^]_i_ increase (Figure 4F and 4G). Collectively, these data suggest that the NQO1-mediated CD38/NAD^+^/cADPR pathway activates the CaMKII/AMPK pathway via accumulation of [Ca^2+^]_i_ under OGD.

### NQO1-mediated activation of AMPK plays a critical role in OGD-induced cell death

**Figure 5:**
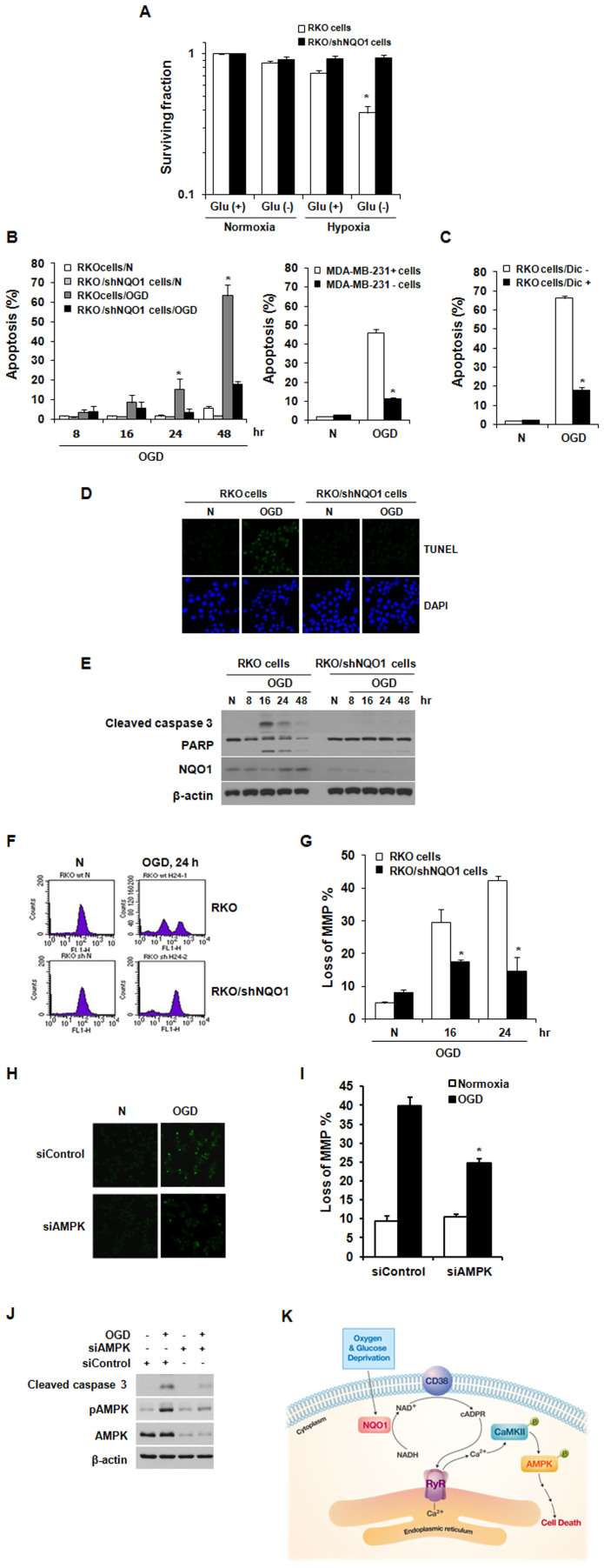
NQO1-mediated AMPK activation leads to OGD-induced cell death. (A) Role of NQO1 in OGD-induced clonogenic cell death. Results from five independent experiments are expressed as means ± SEMs. (B)–(C) Role of NQO1 in OGD-induced apoptosis. The results from three independent experiments are expressed as means ± SEM (*, P < 0.05). (D) Representative photomicrographs of apoptotic (TUNEL-stained) cells exposed to normoxic or OGD conditions for 24 h. DAPI (4',6-diamidino-2-phenylindole) was used to stain nuclei. (E) Involvement of NQO1 in OGD-induced caspase-3 activation and PARP cleavage. RKO and RKO/shNQO1 cells were exposed to normoxic or OGD conditions for the indicated times, and whole-cell extracts were analyzed by immunoblotting using antibodies against Cleaved caspase 3, PARP, NQO1, and β-actin. (F)–(G) Involvement of NQO1 in ODG-induced disruption of mitochondrial membrane potential. RKO and RKO/shNQO1 cells were exposed to normoxic or OGD conditions for the indicated times, and mitochondrial membrane potential was analyzed by flow cytometry using 30 nM DiOC6(3). Representative flow cytometric images (F) and quantification of loss of MMP% (G) are shown. The results from three independent experiments are expressed as means ± SEM (*, P < 0.05). (H) Involvement of AMPK in OGD-induced apoptosis. RKO cells transfected with siRNA against AMPK were exposed to OGD for 24 h. Representative photomicrographs of apoptotic (TUNEL-stained) cells are shown. (I) Involvement of AMPK in the OGD-induced disruption of mitochondrial membrane potential. RKO cells transfected with siRNAs against AMPK were exposed to OGD for 24 h, and mitochondrial membrane potential was analyzed by flow cytometry using 30 nM DiOC6(3). The results from three independent experiments are expressed as means ± SEM (*, P < 0.05). (J) Involvement of AMPK in OGD-induced caspase-3 activation and PARP cleavage. RKO cells transfected with siRNAs against AMPK were exposed to OGD for 8 h, and whole-cell lysates were analyzed by immunoblotting using antibodies against Cleaved caspase 3, PARP, NQO1, and β-actin. (K) Schematic model of how OGD induces cancer cell death via activation of CD38/cADPR/RyR/Ca^2+^/CaMKII/AMPK signaling in NQO1-expressing cancer cells. Abbreviation: N, normoxic control; Dic, Dicumarol.

Previous studies have shown that serum starvation plus extreme (≤0.02% O_2_) or modest (0.5% O_2_) hypoxia results in apoptotic cell death^39^,^40^. Therefore, we investigated the role of NQO1 in OGD-induced cell death. As shown in Figure 5A, the surviving fraction of clonogenic parental RKO cells significantly decreased to 0.38 under OGD, which was far greater than the cell death under the lower-stress conditions of glucose withdrawal or hypoxia alone. Furthermore, RKO/shNQO1 cells were more resistant to OGD conditions compared to parental RKO cells, suggesting that NQO1 is involved in this OGD-induced clonogenic cell death (Figure 5A). We subsequently used propidium iodide (PI) staining and flow cytometry to confirm the role of NQO1 in OGD-induced apoptotic cell death. Exposure of parental RKO cells to OGD for 48 h caused apoptosis in more than 60% of cells, whereas < 20% of RKO/shNQO1 cells was apoptotic (Figure 5B). To confirm these results, we assessed the OGD-induced apoptotic cell death in MDA-MB-231+ and MDA-MB-231- cells. In agreement with the results obtained in RKO cells, NQO1-expressing MDA-MB-231+ cells showed higher apoptotic cell death, compared to parental MDA-MB-231- cells (Figure 5B). To further examine the involvement of NQO1 in the OGD-induced apoptotic cell death, RKO cells were treated with or without dicumarol and exposed to OGD for 48 h. As shown in Figure 5C, dicumarol effectively inhibited the OGD-induced apoptotic cell death. In addition, similar results were obtained using terminal transferase dUTP nick-end labeling (TUNEL) assays (Figure 5D), further confirming that NQO1 plays a role in OGD-induced apoptotic cell death. Studies aimed at testing whether NQO1 was involved in activating caspase pathways under OGD conditions showed that OGD activated caspase-3, induced PARP cleavage, and disrupted mitochondrial membrane potential in parental RKO cells, but not in RKO/shNQO1 cells (Figures 5E–5G). These results clearly indicate that activation of the caspase pathway is essential for OGD-induced cell death in NQO1-expressing cells. Since the role of AMPK in metabolic stress-induced cell death remains controversial^15^,^41^,^42^,^43^, we used AMPK-targeting siRNAs to study its involvement in OGD-induced cell death. Our results revealed that siRNA-mediated knockdown of AMPK significantly attenuated OGD-induced apoptotic cell death in parental RKO cells possessing NQO1 (Figure 5H) and efficiently blocked the OGD-induced disruption of mitochondrial membrane potential and activation of caspase-3 (Figure 5I and 5J). Taken together, these results indicate that AMPK plays a crucial role in OGD-induced cell death.

## Discussion

Here, we investigated the mechanisms underlying OGD-induced cell death. We observed that OGD induces NQO1-mediated activation of cADPR/RyR, increasing [Ca^2+^]_i_ and activating CaMKII. This activates AMPK and subsequently suppresses the mTOR cascade, leading to apoptotic cell death.

NQO1 is highly expressed in cancer cells and has been implicated in cellular defense mechanisms against free radical damage, oxidative stress, inflammatory responses, and carcinogenesis^27^,^28^,^44^,^45^,^46^. At present, the mechanisms underlying these opposing effects of NQO1 on cell survival and death remain controversial. Here, we report that NQO1 mediates OGD-induced cancer cell death (Figure 5). The pro-apoptotic effect of NQO1 under OGD was confirmed by observations of caspase-3 and PARP cleavage, DNA fragmentation, and mitochondrial dysfunction in wild-type NQO1-expressing RKO cells but not NQO1-depleted RKO cells (Figure 5). Previous studies have shown that AMPK is activated in response to cellular stresses that inhibit ATP production (such as nutrient deprivation and hypoxia)^33^ or metabolic dysfunctions that demand increased ATP consumption, both of which are associated with increases in the [AMP]/[ATP] ratio. The mechanism underlying this AMP-mediated activation of AMPK is not yet fully understood, but it is generally believed that upstream kinases (e.g., LKB1 and/or CaMKK β) mediate the allosteric binding of AMP to the γ-subunit of AMPK, thereby facilitating the phosphorylation of threonine-172 in the α-catalytic subunit^47^,^49^. Notably, we herein show that the NQO1-mediated activation of AMPK by OGD depends on CaMKII regardless of the intracellular [AMP]/[ATP] ratio (Figure S2).

A recent study demonstrated that hypoxia-induced increases in [Ca^2+^]_i_ promote CaMKKβ-mediated AMPK activation^9^. Here, we also observed a significant increase in [Ca^2+^]_i_ in NQO1-expressing cells in response to OGD (Figure 2A-2D), and found that this could be abolished by the intracellular Ca^2+^ chelator, BAPTA-AM (Figure 2E and 2F). Elevated [Ca^2+^]_i_ binds to numerous Ca^2+^-sensing proteins, including calbindin, calretinin, troponin, and calmodulin^50^. The Ca^2+^/CaM-regulated signaling proteins include members of the CaMK family^51^. The CaMK-kinase cascade consists of CaMKK and its primary targets CaMKI, CaMKIV, and CaMKII^51^, and CaMKKβ is known to be an upstream kinase of AMPK^10^,^48^. In the present study, we observed that CaMKII also acts as an upstream activator of AMPK in NQO1-expressing cells under OGD (Figure 2). Furthermore, we found that the OGD-induced activation of AMPK was inhibited by knockdown of CaMKII, and observed that downregulation of NQO1 blocked the OGD-induced increase in [Ca^2+^]_i_ and activation of CaMKII and AMPK (Figure 2). Taken together, our results show for the first time that the increase in [Ca^2+^]_i_ induced by OGD triggers the activation of CaMKII, which mediates the activation of AMPK in the context of NQO1 expression.

The Ca^2+^-mobilizing second messenger, cADPR, is generated by ADPR-cyclases and increases [Ca^2+^]_i_ by promoting the release of Ca^2+^ from intracellular ER stores via the binding of cADPR to its receptor, RyR^49^,^51^. Consistent with this, we found that RyR (but not other Ca^2+^ channels) contributed to the OGD-induced Ca^2+^ efflux and activation of the CaMKII/AMPK pathway (Figure 3). NQO1 catalyzes the oxidation of NADH to NAD^+^ using various quinones, leading to elevation of NAD^+^ levels in the cytosol^19^,^52^. Previous studies have shown that NQO1 activity is increased by OGD^26^. Here, we observed that OGD also increases NQO1 activity and the intracellular ratio of NAD^+^ to NADH (Figure 1C and 4A). In turn, this increase of NAD^+^ in NQO1-expressing cells appears to activate the cADPR/RyR pathway, contributing to high [Ca^2+^]_i_ under OGD conditions.

One of the major roles of AMPK is controlling the mTOR-dependent translation process, which requires the integrity of the TSC1/2 inhibitory complex^52^. A recent study using TSC2-deficient MEFs suggested that the AMPK-mediated phosphorylation of raptor is an important metabolic checkpoint that inactivates mTOR^53^. Raptor acts as a scaffold for recruiting and activating mTOR substrates, including S6K and 4E-BP1, and the deregulation of mTOR activity induces hypophosphorylation of S6K and 4E-BP1, suppressing cell proliferation and cell survival^53^. Interestingly, we found that mTOR signaling was consistently inactivated in NQO1-expressing cells (but not in NQO1-depleted cells) under OGD (Figure S2). Moreover, knockdown of AMPK partially recovered the phosphorylation levels of S6K and 4E-BP1 under OGD (Figure S2) and protected NQO1-expressing cells from OGD-induced cell death (Figure 5). In this study, we used RKO and MDA-MB-231 cells. To complement our data for general screen, we investigated the effect of NQO1 on the OGD-induced activation of the CaMKII/AMPK pathway via accumulation of [Ca^2+^]_i_ and also on the increase in apoptotic cell death in HCT116, HeLa and MEF cell lines. In agreement with the results obtained in RKO and MDA-MB-231 cells, down-regulation of NQO1 effectively inhibited OGD-induced Ca^2+^/CaMKII/AMPK and apoptotic cell death (Figure S5). Collectively, these results clearly demonstrate that OGD induces cell death by activating the CD38/cADPR/RyR/Ca^2+^/CaMKII/AMPK signaling pathway in NQO1-expressing cancer cells (Figure 5K).

## Methods

### Cell culture

RKO human colorectal cancer cells carrying wild type NQO1 and shRNA against NQO1 (shNQO1) were maintained in Dulbecco's modified Eagle medium (DMEM) supplemented with 10% (v/v) fetal bovine serum (FBS), penicillin (50 units/ml), and streptomycin (50 μg/ml)^54^. NQO1-deficient parental MDA-MB-231 human breast cancer cells (MDA-MB-231- cells) and MDA-MB-231 cells stably transfected with NQO1 (MDA-MB-231+ cells) were kindly provided by Dr. David Boothman (University of Texas Southwestern Medical Center, Dallas, TX, USA). HCT116, HeLa and MEF cells were maintained in DMEM supplemented with 10% (v/v) fetal bovine serum (FBS), penicillin (50 units/ml), and streptomycin (50 μg/ml). MDA-MB-231 cells were cultured in RPMI-1640 medium supplemented with 10% (v/v) FBS, penicillin (50 units/ml), and streptomycin (50 μg/ml).

### Reagents

Antibodies against CaMKII, pCaMKII, AMPK, pAMPK, mTOR, pmTOR, S6K, pS6K, 4E-BP, ACC, pACC, and Cleaved caspase-3 were obtained from Cell Signaling Technology (Beverly, MA, USA). Antibodies against NQO were purchased from Invitrogen (Carlsbad, CA, USA). Anti-β-actin, anti-rabbit IgG, and anti-mouse IgG antibodies were purchased from Sigma-Aldrich Co. (St. Louis, MO, USA).

### Oxygen and glucose deprivation (OGD)

OGD was obtained using a modification of a previously described procedure^55^. Briefly, cells were seeded in glass culture plates (5 × 10^5^ cells/plate) coated with 0.01% gelatin. After an overnight incubation in a 5% CO_2_-air atmosphere at 37°C, cells were washed three times with phosphate-buffered saline (PBS) and the culture plates were replenished with glucose-free medium containing 10% FBS. The culture plates were flushed for 1h with 5% CO_2_-95% N_2_ to achieve pO_2_ values ≤ 0.01% and then the cells were placed in a 37°C anaerobic chamber (pO_2_ ≤ 0.1%).

### siRNA transfection

siRNA duplexes targeting NQO1 (5′-CAGUACACAGAUACCUUGA-3′) were purchased from Bioneer (Daejeon, Korea). siRNA duplexes targeting AMPK (5′-CGACUAAGCCCAAAUCUUU-3′) were purchased from Dharmacon. siRNA duplexes targeting CaMKII, RyR and CD38 were purchased from Santa Cruz Biotechnology. AccuTarget negative control siRNA (Invitrogen) was used as a negative control. LipofectAMINE 2000 (Invitrogen) was used to transfect siRNA duplexes (final concentration, 50 nM) according to the manufacturer's recommendations. After transfection, cells were processed for immunoblotting and/or assays as indicated.

### Plasmids

To generate pCDNA3.1-myc-his_6_-NQO1 and pCDNA3.1-myc-his_6_-NQO1 C609T mutant, the complementary DNA of NQO1 was obtained from RKO cells using reverse transcription–PCR, annealed and ligated into *Kpn*I- and *Xho*I-digested vector (pCDNA3.1-myc-his_6_). The primer sequences used were as follows: 5′-GGG GTA CCA TGG TCG GCA GAA GAG CAC-3′ (forward), 5′-CCG CTC GAG TTT TCT AGC TTT GAT CTG G-3′ (reverse), 5′-TCT TAG AAT CTC AAC TGA CA-3′ (C609T forward) and 5′-TGT CAG TTG AGA ATT CTT AAG A-3′ (C609T reverse). All constructs were confirmed by DNA sequencing (Bionics, Seoul, Republic of Korea).

### Measurements of cellular AMP and ATP

Acid-soluble extracts of cell suspensions were prepared by adding ice-cold 70% perchloric acid to a final concentration of 7% (wt/vol). Acid-insoluble fractions were removed by centrifugation for 10 min at 13,000 × g and 4°C. Extracts were neutralized with ice-cold 5 M K_2_CO_3_ and stored at -80°C until analyzed. Before analysis by high performance liquid chromatography (HPLC), the potassium perchlorate precipitate was removed by centrifugation, as above. Analyses were performed using a Dionex HPLC system (Dionex, Sunnyvale, CA, USA), which included a PDA-100 photodiode array detector, a GP-50 gradient pump, an AS50 autosampler, and an AS50 thermal compartment.

### Quantification of clonogenic death

The effect of OGD on the clonogenicity of cells was determined. After incubation under OGD for 24h, cells were trypsinized and then different numbers of cells were plated in cell culture flasks and incubated in a 5% CO_2_ incubator at 37°C for 7–10 days. Colonies formed were fixed with methanol and stained with crystal violet (0.1% in methanol); the number of colonies containing more than 50 cells was counted. The surviving cell fractions of treated groups were calculated by expressing the plating efficiency of treated cells relative to that of untreated control cells.

### Quantification of apoptosis

Cells were collected by trypsinization, washed two times with PBS, resuspended in 1 ml PBS containing 0.1% Triton X-100, 0.1 mM EDTA, 10 mg/ml DNase-free RNase A and 2 mg/ml PI, and incubated for 1 h in the dark at 37°C. The percentage of apoptotic cells (sub-G_1_ population) was determined by flow cytometry using a FACSCalibur system (Becton Dickinson, San Jose, CA, USA).

### Measurement of [Ca^2+^]_i_

Cells grown on 8-well Lab-Tek II coverslips were incubated with the Ca^2+^-sensitive dye Fluo-4-AM (5 μM; Molecular Probes, Eugene, OR, USA) for 45 min at 37°C. Cells were then rinsed three times with media and incubated for 30 min in non-supplemented medium to allow complete de-esterification of AM esters. The Fluo-4-loaded cells were then treated with 30 μM BAPTA-AM and exposed to OGD. [Ca^2+^]_i_ was measured from fluorescent images of cells captured 30 min subsequently using a fluorescence microscope (Nikon C1-Plus laser-scanning TE2000-E; Nikon, Tokyo, Japan).

### NAD^+^/NADH Assay

A total of 4 × 10^5^ cells were used in each assay, and intracellular NAD**^+^**/NADH was extracted with 400 μl of NAD**^+^**/NADH extraction buffer by subjecting the cells to two cycles of freeze/thaw (20 min on dry ice followed by 10 min at room temperature). To detect total NAD**^+^** and NADH (NADt), 50 μl of each extracted sample was transferred into a 96-well plate in duplicates. The plate was incubated at room temperature for 5 min in the presence of 100 μl of NAD Cycling Mix to allow the conversion of NAD**^+^** to NADH. To detect NADH, 200 μl of extracted solution was taken from each sample and heated at 60°C for 30 min in a heating block. Under these conditions, all NAD**^+^** was decomposed, whereas NADH remained intact. Then 50 μl of each NADH sample was taken into a 96-well plate in duplicates. Subsequently 10 μl of NADH developing solution was added into each well. After 40 min in the dark, the plates were read at 450-nm wavelength on a microplate reader. The ratio of NAD**^+^**/NADH was calculated as follows: (NADt **-** NADH)/NADH.

### Immunoblot analysis

Cells were lysed; proteins were separated by SDS-polyacrylamide gel electrophoresis (PAGE) and transferred to nitrocellulose membranes. The membranes were blocked with 1% (v/v) nonfat dry milk in Tris-buffered saline with 0.05% Tween20 and incubated with antibodies against proteins of interest. The blots were labeled with goat anti-rabbit or anti-mouse IgG conjugated with horseradish peroxidase, and visualized with chemiluminescence (Pierce, Rockford, IL, USA). All immunoblots presented are representative of at least four separate experiments.

### Measurement of mitochondrial membrane potential

Mitochondrial transmembrane potentials were evaluated by incubating cells with 30 nM 3,3′-dihexyloxacarbocyanine iodide [DiOC6(3)] (Fluka, Deisenhofen, Germany) for 30 min at 37°C. Cells were then harvested, washed with PBS, and re-suspended in 1 ml PBS. Mitochondrial transmembrane potentials were measured using a flow cytometer^54^.

### TUNEL assay

Cells grown on 8-well chamber slides were exposed to OGD. After fixing with 4% (v/v) paraformaldehyde for 15 min, cells were washed with PBS containing 1% (w/v) bovine serum albumin, permeabilized with 0.1% (v/v) Triton-X100, washed with PBS, and incubated for 1 h at 37°C in the dark with an apoptosis detection solution (Apoptosis Detection System kit; Roche Molecular Biochemicals, Mannheim, Germany). In situ-labeled nuclei were observed and photographed using a Nikon C1-Plus laser-scanning TE2000-E confocal microscope.

### Statistical analysis

Microsoft Excel was used for statistical analyses. Differences between two groups were analyzed using Student's *t-*tests, and values of *P* <0.05 (*) were considered significant.

## Author Contributions

H.L. performed the research; H.J.P. supervised and designed parts of the study; M.T.P. and J.S.L. analyzed the data; B.H.C., E.T.O., J.K.L. and H.J.P. wrote the manuscript. All authors reviewed the manuscript.

## Supplementary Material

Supplementary Information Supplementary informations
