# Supplementary material for: NQO1-induced activation of AMPK contributes to cancer cell death by oxygen-glucose deprivation
Source: Sci Rep. 2015 Jan 14;5:7769. doi: 10.1038/srep07769 (PMC4293602; doi:10.1038/srep07769)
Supplement: Supplementary Information — s [file srep07769-s1.pdf]

SUBJECT AREAS: CANCER

NQO1-induced activation of AMPK contributes to cancer cell death by oxygen-glucose deprivation

Hyemi Lee[1,7], Eun-Taex Oh[1,2,7], Bo-Hwa Choi[1,3,7], Moon-Taek Park[1,4], Ja-Kyeong Lee[2,5], Jae-Seon Lee[2,6] and Heon Joo Park[1,2,*]

[1]Department of Microbiology, Inha Research Institute for Medical Sciences, College of Medicine, Inha University, Incheon, Korea, [2]Hypoxia-related Disease Research Center, College of Medicine, Inha University, Incheon, Korea, [3]Pohang Center for Evaluation of Biomaterials, Pohang Technopark, Pohang, Gyeongbuk, Korea, [4]Research Center, Dongnam Institute of Radiological & Medical Sciences (DIRAMS), Busan, Korea, [5]Department of Anatomy, College of Medicine, Inha University, Incheon, Korea, [6]Department of Biomedical Sciences, College of Medicine, Inha University, Incheon, Korea.

*Correspondence and requests for materials should be addressed to H.P. (park001@inha.ac.kr)

[7]These authors contributed equally

Figure S1.

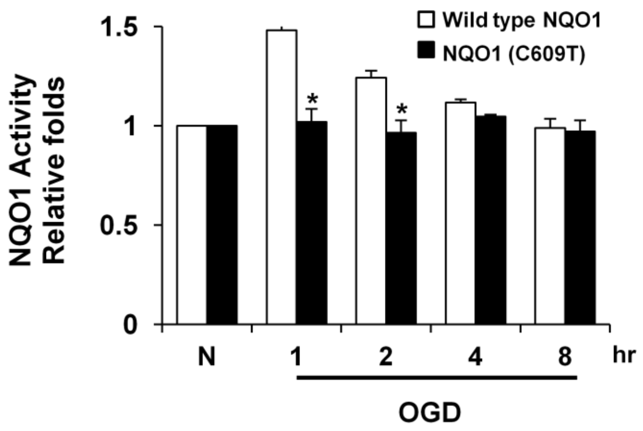
Wild type NQO1
NQO1 (C609T)
NQO1 Activity
Relative folds
1.5
1
0.5
0
*
*
N
1
2
4
8
hr
OGD

Figure S2.

A

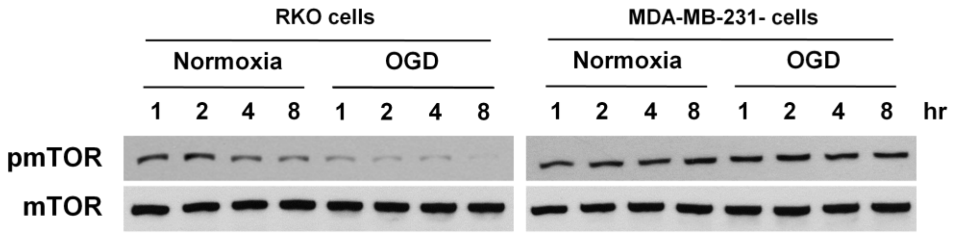
RKO cells
MDA-MB-231- cells
Normoxia
OGD
Normoxia
OGD
1 2 4 8 1 2 4 8 1 2 4 8 1 2 4 8 hr
pmTOR
mTOR

B

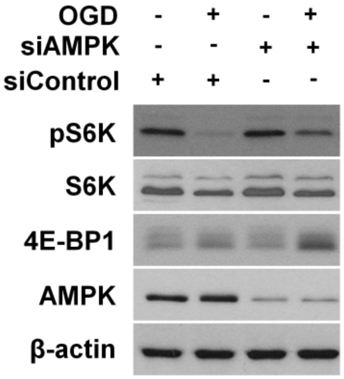
OGD - + - +
siAMPK - - + +
siControl + + - -
pS6K
S6K
4E-BP1
AMPK
β-actin

Figure S3.

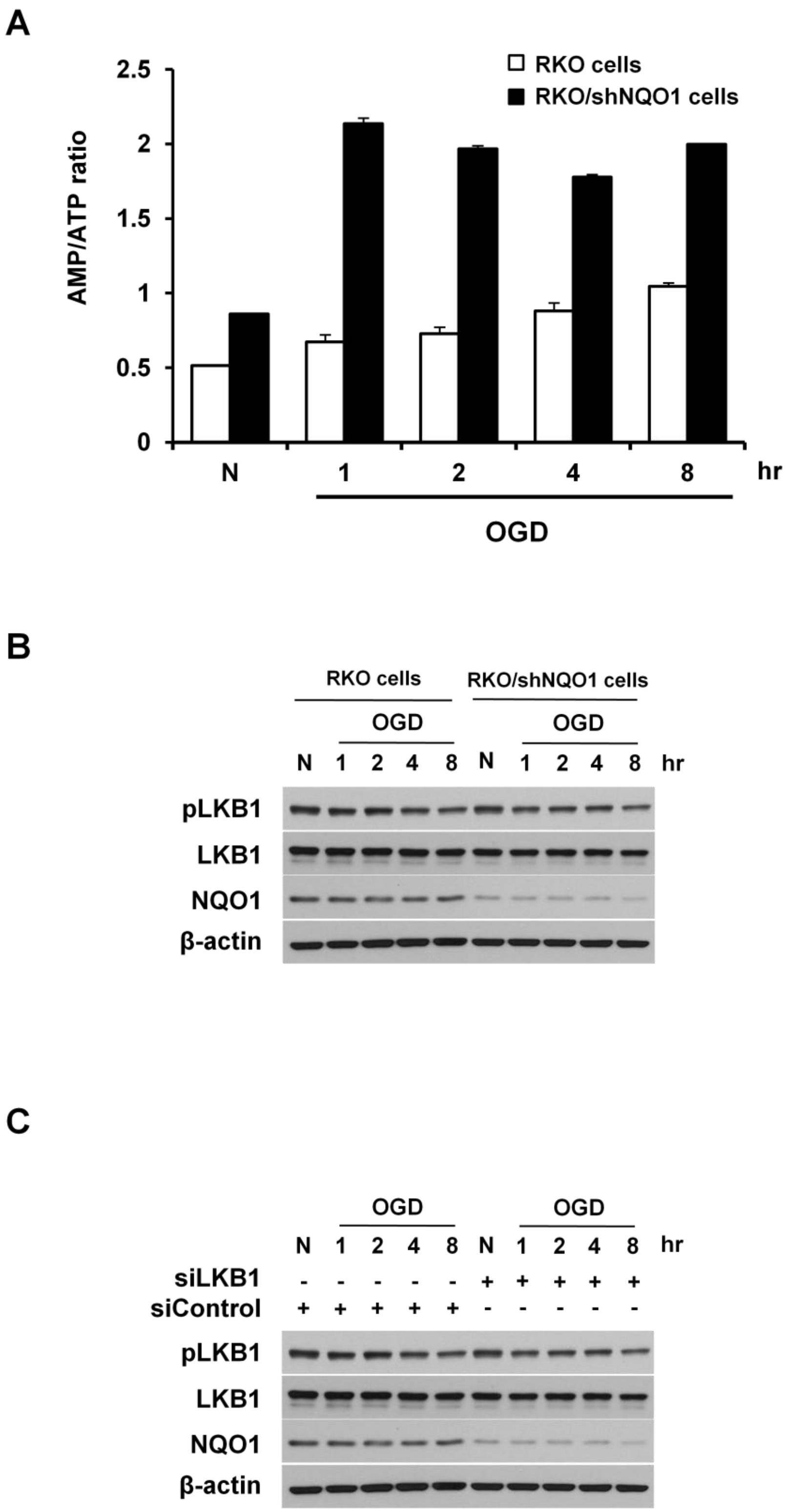
A
RKO cells
RKO/shNQO1 cells
AMP/ATP ratio
2.5
2
1.5
1
0.5
0
N
1
2
4
8
hr
OGD
B
RKO cells
RKO/shNQO1 cells
OGD
OGD
N 1 2 4 8 N 1 2 4 8 hr
pLKB1
LKB1
NQO1
β-actin
C
OGD
OGD
N 1 2 4 8 N 1 2 4 8 hr
siLKB1 - - - - - + + + + +
siControl + + + + + - - - - -
pLKB1
LKB1
NQO1
β-actin

Figure S4.

A

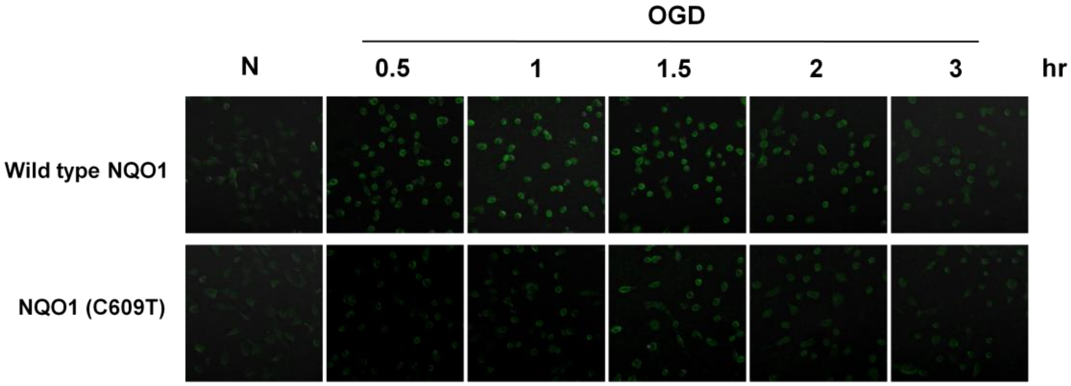
OGD
N
0.5
1
1.5
2
3
hr
Wild type NQO1
NQO1 (C609T)

B

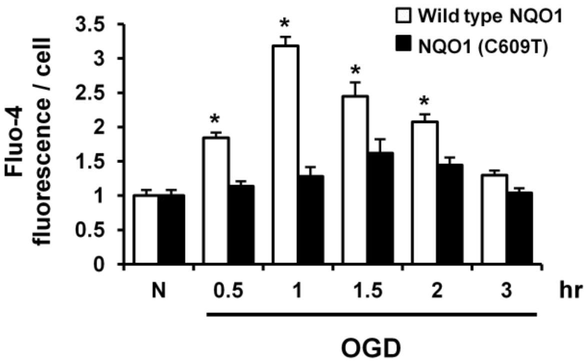
Fluo-4 fluorescence / cell
3.5
3
2.5
2
1.5
1
0.5
0
Wild type NQO1
NQO1 (C609T)
*
N
0.5
1
1.5
2
3
hr
OGD

Figure S5.

A

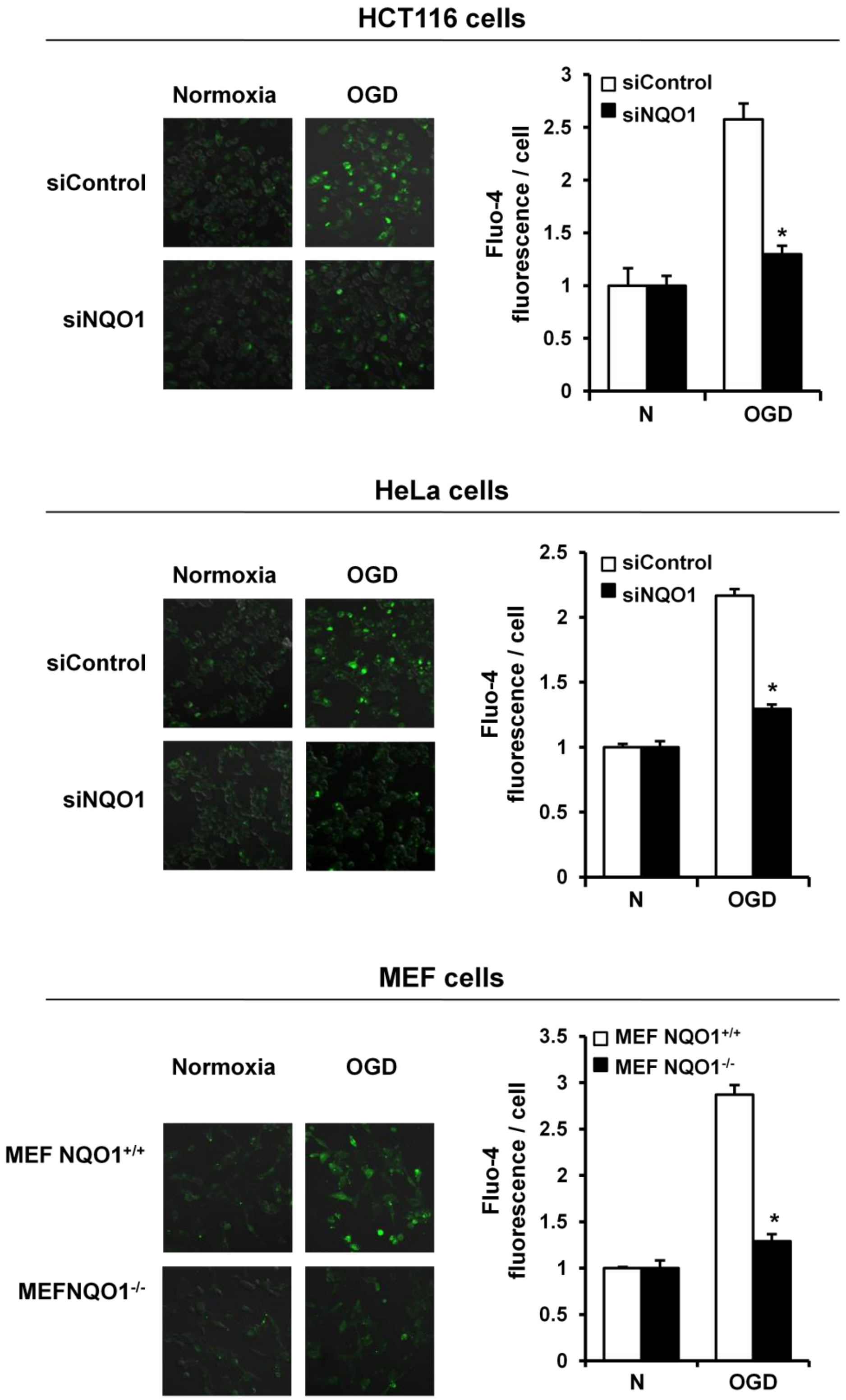
HCT116 cells
Normoxia
OGD
siControl
siNQO1
Fluo-4 fluorescence / cell
3
2.5
2
1.5
1
0.5
0
N
OGD
*
HeLa cells
Normoxia
OGD
siControl
siNQO1
Fluo-4 fluorescence / cell
2.5
2
1.5
1
0.5
0
N
OGD
*
MEF cells
Normoxia
OGD
MEF NQO1+/+
MEFNQO1-/-
MEF NQO1+/+
MEF NQO1-/-
Fluo-4 fluorescence / cell
3.5
3
2.5
2
1.5
1
0.5
0
N
OGD
*

Figure S5. continued

B

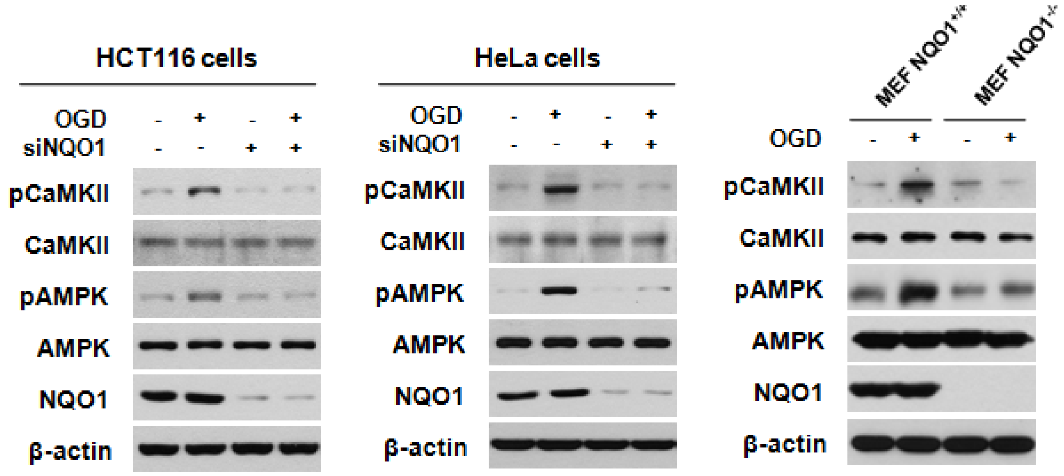

C

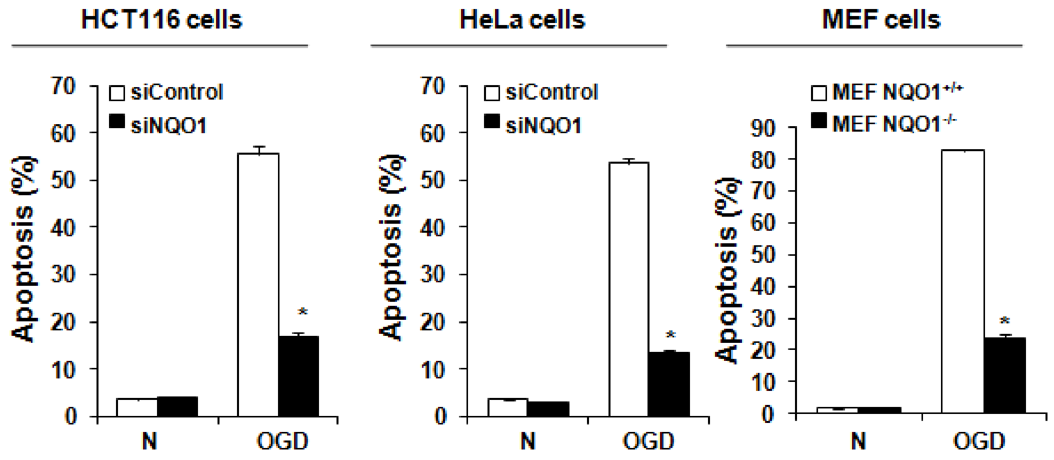

Supplementary figure caption

Figure S1

Activities of wild-type and mutant (C609T) NQO1 under OGD. MDA-MB231-/pNQO1 and MDA-MB231-/pNQO1 (C609T) cells were exposed to OGD for the indicated times, and NQO1 activity was assessed. The results from three independent experiments are expressed as means ± SEM (*, $P < 0.05$).

Figure S2

Involvement of NQO1 in OGD-induced AMPK/mTOR signaling. (A) Expression levels of pmTOR and mTOR in parental RKO and MDA-MB-231- cells exposed to OGD for the indicated times. (B) RKO cells transfected with siRNAs against AMPK were exposed to OGD for 8 h, and the phosphorylation levels of S6K, S6K, 4E-BP1 and AMPK in whole-cell lysates were determined by immunoblotting.

Figure S3

OGD does not act through LKB1 to activate AMPK. (A) Intracellular [AMP]/[ATP] ratios in RKO and RKO/shNQO1 cells exposed to OGD for the indicated times. (B) Expression of pLKB, LKB, and NQO1 in RKO and RKO/shNQO1 cells exposed to OGD for the indicated times. (C) Effect of LKB1 knockdown on the expression levels of pAMPK and pACC in RKO cells. Abbreviation: N, normoxic control.

Figure S4

Effect of OGD on $[Ca^{2+}]_i$ in MDA-MB231-/pNQO1 and MDAMB231-/pNQO1 (C609T) cells (A)-(B) Fluo-4-AM-loaded cells were exposed to OGD for the indicated times. Representative fluorescence microscopic images (A) and quantification of $[Ca^{2+}]_i$ (B) are shown. The results from three independent experiments are expressed as means ± SEM (*, $P < 0.05$). Abbreviation: N, normoxic control.

Figure S5

Effect of NQO1 on OGD-induced activation of CaMKII/AMPK signaling pathway via increase of $[Ca^{2+}]_i$ and cancer cell death in various cell lines. (A) Fluo-4-AM-loaded cells were exposed to OGD for 1 h. Representative fluorescence microscopic images and quantification of $[Ca^{2+}]_i$ are shown. The results from three independent experiments are expressed as means ± SEM (*, $P < 0.05$). (B) The cells were exposed to OGD for 1 h, and whole-cell lysates were analyzed by immunoblotting using antibodies against pCaMKII, pAMPK, CaMKII and AMPK. (C) Role of NQO1 in OGD-induced apoptosis. The cells were exposed to OGD for 48 h, and the apoptotic cells were analyzed using flow cytometry. The results from three independent experiments are expressed as means ± SEM (*, $P < 0.05$). Abbreviation: N, normoxic control.
